# Electronic Structure of the Complete Series of Gas-Phase Manganese Acetylacetonates by X-ray Absorption Spectroscopy

**DOI:** 10.1021/acs.jpca.3c02794

**Published:** 2023-08-17

**Authors:** Olesya S. Ablyasova, Meiyuan Guo, Vicente Zamudio-Bayer, Markus Kubin, Tim Gitzinger, Mayara da Silva Santos, Max Flach, Martin Timm, Marcus Lundberg, J. Tobias Lau, Konstantin Hirsch

**Affiliations:** † Physikalisches Institut, Albert-Ludwigs-Universität Freiburg, Hermann-Herder-Str. 3, 79104 Freiburg, Germany; ‡ Abteilung für Hochempfindliche Röntgenspektroskopie, Helmholtz-Zentrum Berlin für Materialien und Energie, Albert-Einstein-Str. 15, 12489 Berlin, Germany; § SSRL, SLAC National Accelerator Laboratory, Menlo Park, California 94025, United States; ∥ Department of Chemistry-Ångström Laboratory, Uppsala University, SE-75120 Uppsala, Sweden

## Abstract

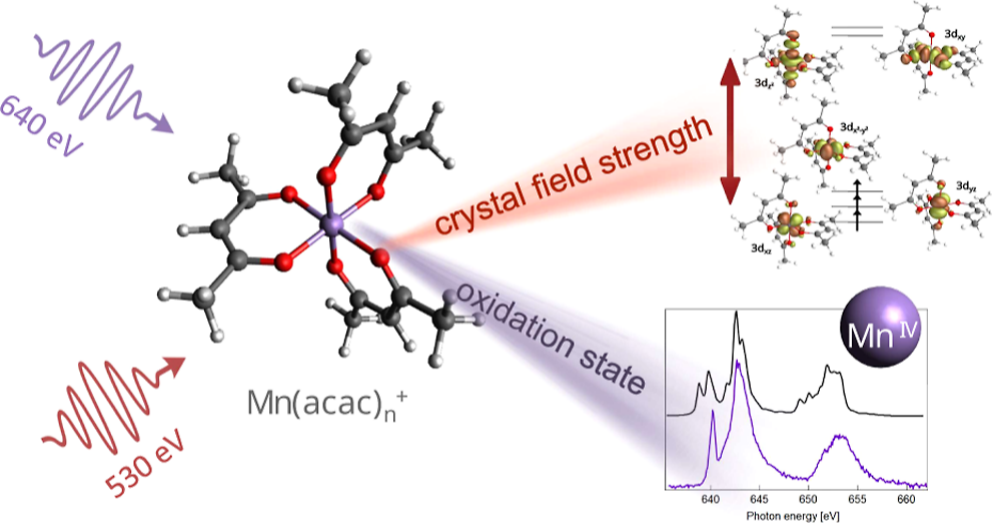

Metal centers in transition metal–ligand complexes occur in a variety of oxidation states causing their redox activity and therefore making them relevant for applications in physics and chemistry. The electronic state of these complexes can be studied by X-ray absorption spectroscopy, which is, however, due to the complex spectral signature not always straightforward. Here, we study the electronic structure of gas-phase cationic manganese acetylacetonate complexes Mn(acac)_1–3_^+^ using X-ray absorption spectroscopy at the metal center and ligand constituents. The spectra are well reproduced by multiconfigurational wave function theory, time-dependent density functional theory as well as parameterized crystal field and charge transfer multiplet simulations. This enables us to get detailed insights into the electronic structure of ground-state Mn(acac)_1–3_^+^ and extract empirical parameters such as crystal field strength and exchange coupling from X-ray excitation at both the metal and ligand sites. By comparison to X-ray absorption spectra of neutral, solvated Mn(acac)_2,3_ complexes, we also show that the effect of coordination on the L_3_ excitation energy, routinely used to identify oxidation states, can contribute about 40–50% to the observed shift, which for the current study is 1.9 eV per oxidation state.

## Introduction

There is a longstanding interest in transition-metal acetylacetonate (acac) complexes that has been renewed in recent years due to promising applications in redox flow batteries,^1,2^ industrial aqueous synthesis of metal–organic-frameworks,^3^ and as possible single-molecule magnets^4^ even showing spin crossover behavior in related transition-metal complexes.^5^ Furthermore, transition-metal complexes are relevant for catalysis^6,7^ while the attractiveness of transition-metal acetylonates in particular is based on the ability to chemically control their redox activity by coordination or de-coordination of the metal center.^8^ Among the 3d transition metals, manganese is well known as the main driver of the catalytic water splitting reaction mediated by the oxygen-evolving complex^9−13^ and is relevant as a catalyst in other chemical synthesis reactions as it has access to a variety of oxidation states ranging from −3 to +7.^14−16^ Thus, via the interplay of coordination and oxidation states of the manganese atom the properties of manganese acetylacetonate complexes can be tailored and controlled.

The neutral Mn(acac)_2,3_ molecules in solution were successfully investigated earlier,^17−19^ but the singly coordinated Mn(acac)_1_ has never been observed in solution. Here, we show that the low-coordinated Mn(acac)_1_^+^ complex can be accessed in the gas phase. We are therefore able to investigate the complete range of coordination of cationic Mn(acac)_*n*_^+^, *n* = 1–3 using X-ray absorption spectroscopy (XAS). By employing XAS to the metal center and the ligand we obtain complementary information on the electronic structure of the system.^20,21^ We also perform time-dependent density functional theory (TD-DFT) calculations at the oxygen and carbon K-edges and restricted active space (RAS) calculations accompanied by charge transfer multiplet (CTM) simulations at the manganese L_2,3_ edges. This enables us to get an in-depth insight into the electronic structure of Mn(acac)_*n*_^+^, *n* = 1–3.

## Methods

### Experimental Details

Experiments were performed at the Ion Trap endstation^22,23^ at beamline UE52 PGM of the synchrotron radiation facility BESSY II operated by the Helmholtz-Zentrum Berlin. Electrospraying-solvated Mn(acac)_3_ molecules generates gas-phase cationic Mn(acac)_1–3_^+^ by successive removal of the acetylacetonate ligands depending on spray conditions and ion funnel settings. Two solutions were prepared using 1 mg (560 μg) of Mn^III^(acac)_3_ solvated in 5 mL (6.65 mL) acetonitrile resulting in a concentration of 0.57 mM (0.24 mM) both giving the same results. The molecule of interest is accumulated in a liquid helium-cooled linear Paul trap after selection using a quadrupole mass filter. The mass spectrum of selected Mn(acac)_*n*_^+^ complexes, *n* = 1–3, is shown in Supporting Information, Figure S8. A monochromatic X-ray beam excites the sample along the trap axis. X-ray absorption by the ions is followed by an Auger cascade leading to dissociation of the molecule due to Coulomb explosion. X-ray absorption is measured in ion yield mode by detecting photo fragments (listed in Supporting Information, Table S4) using a time-of-flight mass spectrometer. Energy resolution and step size at the different absorption edges are given in Supporting Information, Table S2. Photon energy calibration was performed using neon K-edge photoionization in the beamline ionization cell^24^ and checked at the oxygen K-edge, giving a photon energy uncertainty of ±0.1 eV.

### Computational Details

Geometry optimization of all complexes was performed with Gaussian 09. E01,^25^ using DFT employing the B3LYP functional^26,27^ and 6-31G(d) basis set.^28^ Cartesian coordinates of all ground-state structures are given in the Supporting Information. For Mn(acac)_2_^+^, two structures, a planar and a distorted square planar, appeared close in energy. For these two structures, relative stabilities were calculated with B3LYP/6-311+G(2df,2pd)^28^ as well as CASPT2^29^/ANO-RCC-VTZP.^30^ The CASPT2 calculations were performed with OpenMolcas v18.09.^31^

The carbon and oxygen K-edge XAS were calculated with the ORCA 4.1.2 software package using TD-DFT and employing B3LYP/6-31G(d) functional and basis set. The calculated spectra were broadened with Gaussian functions with FWHM = 0.6 eV and shifted in energy as listed in Supporting Information, Table S3 for carbon K-edge and oxygen K-edge XAS to match the energies of the experimental spectra.

The manganese L_2,3_ edge XA spectra were calculated using the RAS approach^32−34^ in OpenMOLCAS v18.09 with RASPT2^35^/ANO-RCC-VDZP using a minimal active space of five metal 3d character orbitals. These orbitals were placed in the RAS2 space where all possible excitations were allowed. The manganese 2p orbitals were placed in the RAS1 space, allowing a maximum of one excitation. Tests of RAS spectral sensitivity shows that good spectral quality can be achieved with relatively small basis sets and that many systems with weak ligands can be described without the use of large active spaces.^36,37^ Core hole states were generated using a projection operator that selectively removes configurations with fully occupied core orbitals.^38,39^ Core-excited states with Δ*S* = 0, ±1 relative to the ground state were included in the calculations to satisfy the spin-selection rules of the electric dipole and the spin–orbit operator. Orbital optimization was performed using state-average RASSCF, performed separately for each spin multiplicity and irreducible representation. All possible states of each spin multiplicity were included in these calculations, using an efficient configuration interaction algorithm to converge the state-average calculations.^40^ To avoid orbital rotation, i.e., that the hole appears in a higher-lying orbital, the core orbitals have been frozen in the final states. The Douglas–Kroll–Hess Hamiltonian is used to describe scalar relativistic effects.^41,42^ RASPT2 calculations were performed with the default ionization potential electron affinity shift of 0.25 hartree (6.8 eV) and an imaginary shift of 0.3 hartree (8.2 eV). Results for calculations with different imaginary shift are shown in Supporting Information, Figure S3. Spin–orbit coupling is described by a RAS state-interaction (RASSI) approach.^43,44^ The calculated spectra were convoluted with a Lorentzian of 0.34 eV full width at half-maximum (FWHM) for the L_3_ edge, and 0.39 eV FWHM for the L_2_ edge, to account for core hole lifetime broadening.^45^ The experimental broadening is simulated with an additional Gaussian broadening of 0.2 eV (FWHM). Calculated spectra were shifted in energy to align with the experimental spectra, see the Supporting Information for details.

Crystal field (CF) and CTM simulations were performed using the CTM4XAS 5.5 software package.^46^ Parameters employed for simulating L_2,3_ edge spectra of Mn(acac)_*n*_^+^, *n* = 1–3, are given in Supporting Information, Table S1. Simulated spectra were shifted in energy to match the experimental spectra as given in Supporting Information, Table S3. Both Lorentzian (0.34 eV FWHM for L_3_ and 0.39 eV FWHM for L_2_) and Gaussian broadening (0.2 eV FWHM) was applied^45^ while Slater integrals have been scaled to 80% of their atomic value. CF calculations were performed for a broad range of CF strength, varying parameters 10*Dq*, *Dt*, and *Ds* for their respective symmetries.^47^

## Results and Discussion

### Ground-State Structures

**Figure 1:**
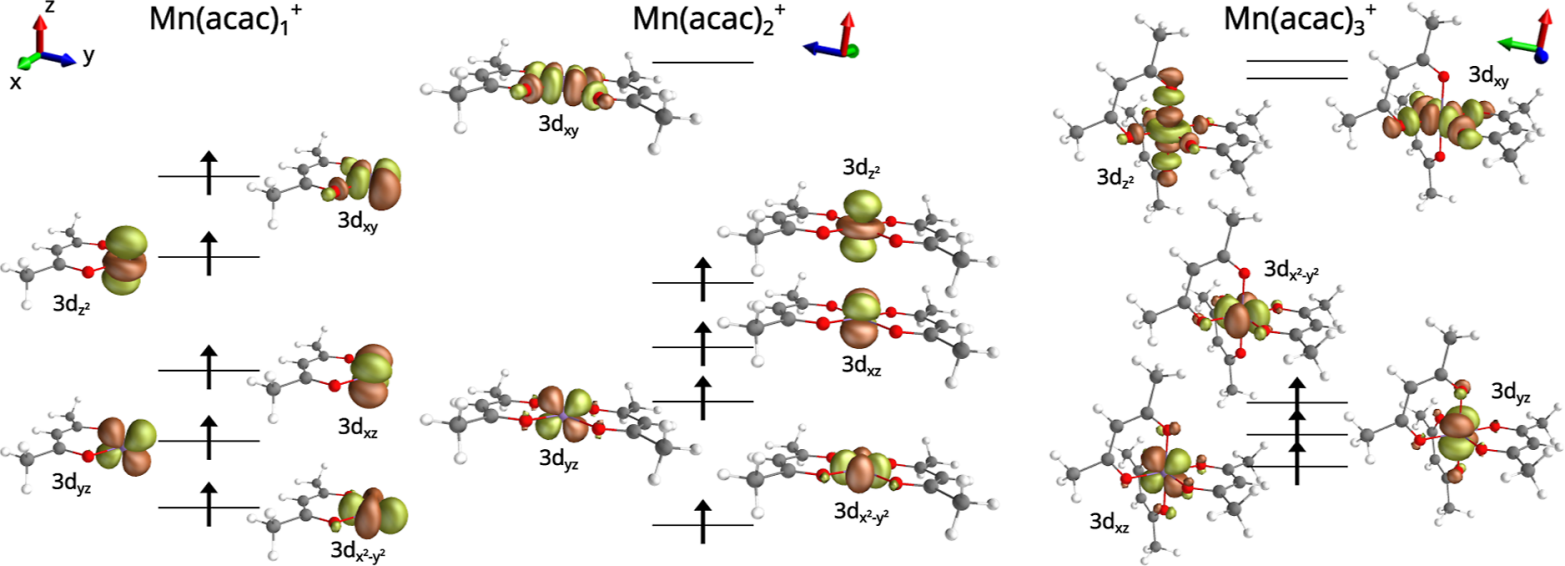
Ground-state structures of Mn(acac)_1–3_^+^ complexes. Additionally shown are isosurface plots of the RAS valence orbitals. Occupied orbitals are indicated by an ↑.

The DFT-optimized ground-state structure of Mn(acac)_1_^+^ is shown in Figure 1. The molecule adopts a C_2v_ symmetry identical to the prediction of isoelectronic Cr(acac)_1_.^48^ There are, however, slight differences in transition-metal–oxygen bond lengths that become shortened in Mn(acac)_1_^+^ (1.900 Å) compared to Cr(acac)_1_ (2.015 Å). Since the transition-metal monoacetylonates have not been stabilized in the condensed phase or as solvated species, there is no experimental data on the structure of Mn(acac)_1_^+^.

For Mn(acac)_2_^+^, a square planar and a slightly distorted square planar structure are almost degenerate with an energy separation of 0.2 kcal/mol, with the former slightly favored by DFT (B3LYP) and the latter slightly favored by CASPT2. Considering the small energy differences, these calculations do not provide a definitive assignment of the structure. In the following, we will discuss the square planar structure, also shown in Figure 1, that is in line with the ground-state structure of isoelectronic Cr(acac)_2_^48,49^ and proposed planar structure for other 3d^4^ metal–ligand complexes.^50^ Changes in the electronic structure of both geometries are minor, and results for the distorted structure are shown in the Supporting Information. However, the structure is distinctively different from bulk, solvated, and neutral gas-phase Mn(acac)_2_ that is a three-dimensional structure of tetrahedral symmetry.^50−53^

Finally, in the fully coordinated Mn(acac)_3_^+^ the Mn central atom is locally octahedrally coordinated, a ball-and-stick model is shown in Figure 1. While the neutral Mn(acac)_3_ is Jahn-Teller distorted resulting in a tetragonally elongated octahedral form,^4,50,54^ the cationic species shows an octahedrally coordinated Mn central atom, which is expected as there is no energy gain in Jahn-Teller distortion with the central Mn atom in oxidation state +4 as will be discussed in detail later.

### Electronic Structure of Mn(acac)_*n*_^+^ Deduced from Mn L_2,3_ Edge X-ray Absorption Spectroscopy

**Figure 2:**
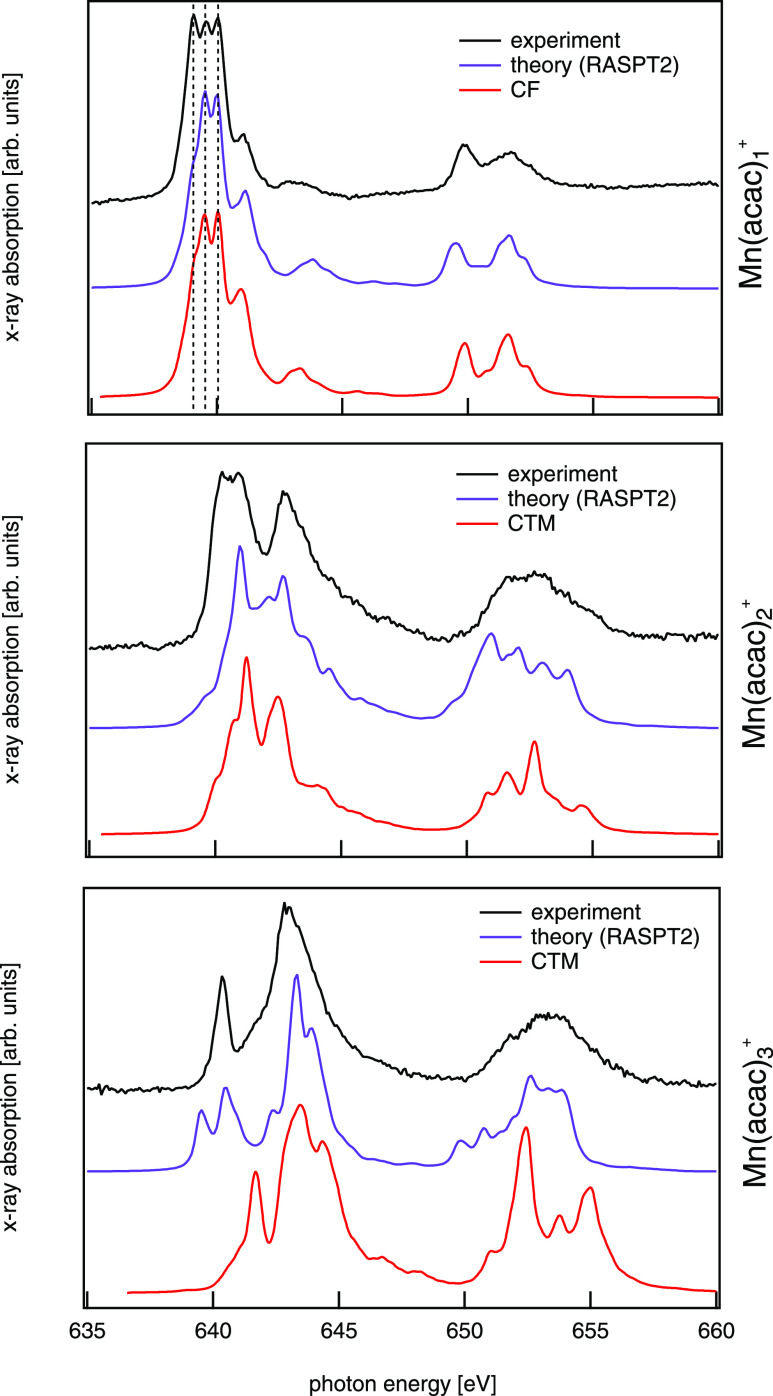
X-ray absorption spectra of gas-phase Mn(acac)_*n*_^+^, *n* = 1–3 at the Mn L_2,3_ edges alongside RASPT2 calculations and CF/CTM simulations. From the RASPT2 calculations, 3d^5^, 3d^4^, and 3d^3^ local configurations of the Mn center were extracted for *n* = 1–3, respectively. Parameters of the CF/CTM calculations are given in Supporting Information, Table S1.

Experimental manganese L_2,3_ edge spectra of Mn(acac)_*n*_^+^, *n* = 1–3 are presented in Figure 2 along with RASPT2 calculations^35^ and CTM simulations.^46^ Both ab initio and semi-empirical fitting models have been shown to give consistent descriptions of the coupling between spectra and electronic structure.^34,55^ There is an overall satisfactory agreement between experimental spectra and RASPT2 calculations highlighting that the applied theory is capable of capturing the correlated nature of the electronic structure of Mn(acac)_*n*_^+^, *n* = 1–3.^48,56^ Furthermore, from a comparison of the experimental spectra to the CF/CTM simulations empirical system parameters such as the CF strength parameter 10*Dq* can be extracted.

The spectrum of Mn(acac)_1_^+^ is dominated by an almost atomic-like multiplet structure of the manganese metal center adopting a local 3d^5^ electronic configuration.^57−59^ There is, however, a slight splitting of the leading line at the L_3_ edge (at about 639.5 eV) also indicated by the dashed lines in Figure 2 that can be traced back to the CF splitting in a C_2v_ symmetry caused by the acetylacetonate ligand pushing the 3d_*xy*_ orbital higher in energy as can be seen from a comparison to the CF simulation in Figure 2. In Supporting Information, Figure S4, we show a systematic study of the spectral changes as a function of CF strength and find that a CF strength 10*Dq* of −1.2 eV reproduces the spectrum of Mn(acac)_1_^+^ complex best. The complete set of parameters is given in Supporting Information, Table S1. Also, the RASPT2 calculation reproduces the experimental spectrum reasonably well and finds a mono-configurational, high-spin ^6^A_1_ (C_2v_, a_1_^1^a_1_^1^a_2_^1^(3d_*xy*_)b_1_^1^(3d_*xz*_)b_2_^1^(3d_*yz*_)a_1_^0^(4s)) ground state adopting the same multiplicity as the isoelectronic neutral Cr(acac)_1_ complex but with a different distribution of the electrons among 3d and 4s derived states resulting in a ^6^B_2_ (C_2v_, a_1_^1^a_1_^1^a_2_^1^(3d_*xy*_)b_1_^1^(3d_*xz*_)b_2_^0^(3d_*yz*_)a_1_^1^(4s)) ground state for neutral Cr(acac)_1_.^48^ Note that the electronic configuration of Mn(acac)_1_^+^ does not change when adding the 4s orbital to the active space. The mono-configurational nature and the fact that the experimental spectrum can be reasonably well reproduced by a CF simulation only, implies that there is little ligand–metal or metal–ligand charge transfer and hybridization. Moreover, in Mn(acac)_1_^+^ we find a DFT and RAS Mulliken spin density at the Mn atom of 4.83 and 4.92, respectively. Since the Mulliken spin is known to correlate strongly with the Mn oxidation state,^60^ our results indicate an oxidation state of +2, which is in line with the findings discussed above.

The spectrum of Mn(acac)_2_^+^ can neither be characterized as dominated by atomic multiplets nor by CF splitting making simulation of the spectrum using a CF approach rather challenging. We were, however, able to reasonably well reproduce the L_3_ edge of Mn(acac)_2_^+^ by simulating the spectrum in a simplified square planar symmetry using a CF strength of 2.4 eV and employing charge transfer, which is consistent with the RAS result of orbital energetic ordering. Moreover, from the RASPT2 calculations we can determine the ground state to be a high-spin ^5^A (C_2_) state with four occupied, localized 3d orbitals, which corresponds to an oxidation state of +3 of the manganese metal center. This is further substantiated by a DFT and RAS Mulliken spin population at the Mn atom of 3.99 and 3.90, respectively. This is also in line with isoelectronic Cr(acac)_2_ in ^5^B_1g_ (D_2h_) ground state, where four 3d electron state-derived orbitals are singly occupied.

Interestingly neutral Mn^III^(acac)_3_ and cationic Mn^III^(acac)_2_^+^ can be both approximated as high-spin 3d^4^ systems in a (pseudo-)tetragonal square planar symmetry^4,50,54,61,62^ and might result in similar spectral shapes at the manganese L_2,3_ edges. Indeed, the magnitude of the splitting of the L_3_ line of 1.7 eV is comparable in neutral Mn(acac)_3_^63,64^ and cationic Mn(acac)_2_^+^ (see Supporting Information, Figure S10). Still, there are distinct differences in the spectral shape, while the mean excitation energy is similar as discussed in the next section. Although the effect of changing the geometry within a defined oxidation state has been explored previously, highlighting a redistribution of spectral weight without inducing larger energetic shifts,^63,65^ we speculate that the observed spectral differences between Mn^III^(acac)_3_ and Mn^III^(acac)_2_^+^ are due to a re-ordering of the 3d orbitals induced by the additional ligand in *z*-direction in Mn(acac)_3_. The additional ligand results in an energy penalty for the  orbital pushing it up in energy and switching the order with other occupied 3d orbitals. Again, both neutral Mn^III^(acac)_3_ and cationic Mn^III^(acac)_2_^+^ exist in high-spin 3d^4^ configuration, but the different order of the same occupied orbitals changes the spectral shape.

Finally, a comparison of the experimental manganese L_2,3_ edge spectrum and the RAS/CTM calculations of the fully coordinated Mn(acac)_3_^+^ complex is shown in Figure 2. The spectrum is dominated by CF splitting and the spectral features strongly resemble other Mn^IV^ species in bulk in O_h_ symmetry.^66^ However, to achieve an even better agreement between the experimental and CF/CTM simulated spectrum it is necessary to involve finite hybridization via charge transfer similar to the case of isoelectronic Cr(acac)_3_.^65^ The CF strength parameter 10*Dq* of 2.1 eV extracted from the simulations indicates a high-spin state^59^ and agrees well with values deduced from UV–vis experiments of 2.1 eV^67^ and 2.4 eV^68^ for neutral Mn(acac)_3_. These findings are consistent with our RASSCF calculations that predict a high-spin ^4^A (C_2_) ground state in agreement with isoelectronic Cr(acac)_3_.^48^ Hence, Mn(acac)_3_^+^ is shown to be in oxidation state +4 further evidenced by DFT and RAS Mulliken spin populations of 3.03 and 2.69, respectively, at the Mn center.

As a summary, the CF/CTM simulations indicate that metal–ligand hybridization is negligible in Mn(acac)_1_^+^ while playing an increasing role in Mn(acac)_2,3_^+^ as expected from the increasing oxidation state of the metal.^69^ We also identified the oxidation states of the manganese atoms for all the complexes by comparison to CF/CTM and RAS calculations as well as by analyzing Mulliken spin populations, also matching the formal oxidation states.

### Comparison of L_3_ Excitation Energy Shifts in Cationic and Neutral Mn(acac)_*n*_^+,0^

**Figure 3:**
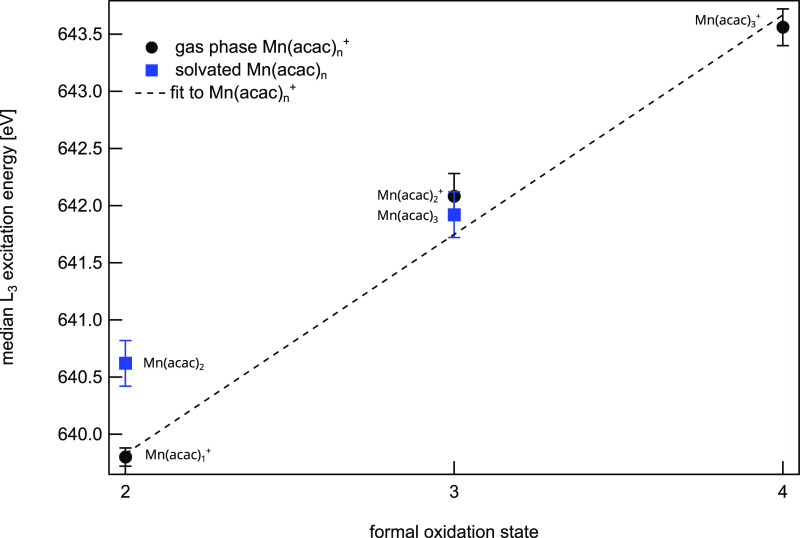
Experimentally determined median L_3_ excitation energy as a function of formal oxidation state for gas-phase Mn(acac)_1–3_^+^ (this study) and solvated, neutral Mn(acac)_2,3_ (extracted from ref (70)). The linear fit to the gas-phase data gives a slope of 1.9 ± 0.1 eV per oxidation state and an offset of 636.2 ± 0.2 eV, respectively.

Analyzing L_3_ excitation energy shifts is a well-established tool to infer oxidation states from L_2,3_ edge XAS.^66,71,72^ Experimentally determined median L_3_ excitation energies of Mn(acac)_*n*_^+^, *n* = 1–3, are shown in Figure 3 as a function of formal oxidation states. As expected they scale linearly, further substantiating our oxidation state assignments from analyzing spectral shapes at the manganese L_2,3_ edge. The extracted slope of 1.9 ± 0.1 eV per oxidation state is well within the range of reported values for Mn ranging from 0.8–2 eV.^66,71−74^ Interestingly, the shift for Mn(acac)_*n*_^+^ complexes, *n* = 1–3, is significantly larger than the reported shift of 0.95 ± 0.02 eV for cationic manganese oxide clusters,^74^ which might be attributed to the different bond character in Mn(acac)_1–3_^+^ and MnO_1–4_^+^. Additionally, we show the median manganese L_3_ excitation energy as a function of oxidation state for neutral Mn(acac)_*n*_^0^ complexes, *n* = 2, 3.

RASPT2 calculations of the oxidation shift of Mn complexes have previously shown deviations of no more than 0.3 eV per oxidation state.^63,74^ Here, the presence of intruder states in some RASPT2 calculations, especially for Mn(acac)_3_^+^, results in a strong dependence of edge position on the choice of imaginary shift, see Supporting Information, Figure S3, which prevents an accurate prediction of the oxidation state shift. At the RASSCF level, the oxidation state shifts are predicted with a deviation of 0.5 eV per oxidation state.

There is a striking difference in the L_3_ excitation energy of 0.8 eV for oxidation state +2 when comparing neutral Mn^II^(acac)_2_ and cationic Mn^II^(acac)_1_^+^. The shift toward higher excitation energy could be induced by a reduced fractional 3d occupation in Mn^II^(acac)_2_ still representing the same oxidation state of +2.^75^ However, in both cases the X-ray absorption spectrum at the Mn L_2,3_ edges could reasonably well be reproduced by CF simulations only and DFT calculations give the same Mulliken spin population^63^ indicating the same or at least very similar 3d occupation in both cases. It previously has been shown that the excitation energy systematically decreases within one oxidation state when decreasing the average coordination of the metal center.^76^ A change in excitation energy of the order of 0.25 eV per change in coordination number has been reported.^76^ Since the coordination number of neutral Mn^II^(acac)_2_ and cationic Mn^II^(acac)_1_^+^ differs by two, we attribute the observed shift to the different coordination of the metal center in both species.

On the other hand, the same L_3_ excitation energy is observed for cationic and neutral Mn(acac)_*n*_^0,+^ in oxidation state +3. As discussed in the previous section, despite the fact that the metal centers in Mn^III^(acac)_3_ and Mn^III^(acac)_2_^+^ exist in nominal different symmetries, the strong Jahn–Teller distortion pushing two of the oxygen atoms away from the metal center resulting in an effective lowering of the coordination number to four, the same as in Mn^III^(acac)_2_^+^ thereby resulting in very similar L_3_ excitation energies.

We want to emphasize that the variation in L_3_ excitation energy due to varying coordination, although sizable, is a second-order effect and the shift is still dominated by the oxidation state of the metal center in these high-spin states.

### Oxygen K-Edge Spectroscopy and Energetic Ordering of the 3d Orbitals

**Figure 4:**
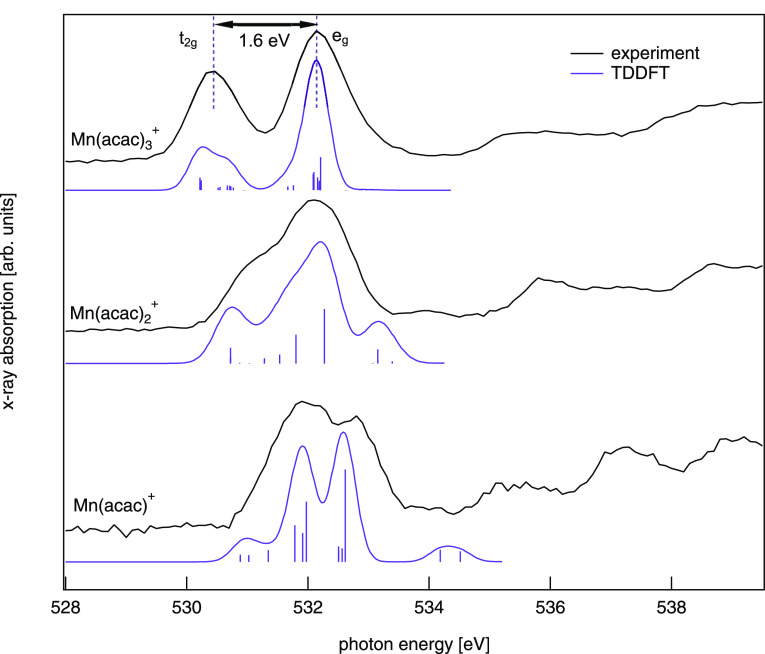
X-ray absorption spectra of gas-phase Mn(acac)_*n*_^+^, *n* = 1–3, at the oxygen K-edge alongside TD-DFT calculations.

In order to get a more complete picture of the electronic structure of Mn(acac)_*n*_^+^, *n* = 1–3, we complement the measurements at the manganese L_2,3_ edges with oxygen and carbon K-edge spectroscopy. In Figure 4 X-ray absorption spectra of gas-phase Mn(acac)_*n*_^+^, *n* = 1–3, at the oxygen K-edge are shown. The experimental X-ray absorption spectra are compared to theoretical spectra from TD-DFT calculations. Low-energy transitions are reproduced well, while high-energy transitions are known to be less well reproduced due to approximations employed in TD-DFT.^77^ Hence, we are focusing the analysis to the low-energy region between 530 and 534 eV.

**Figure 5:**
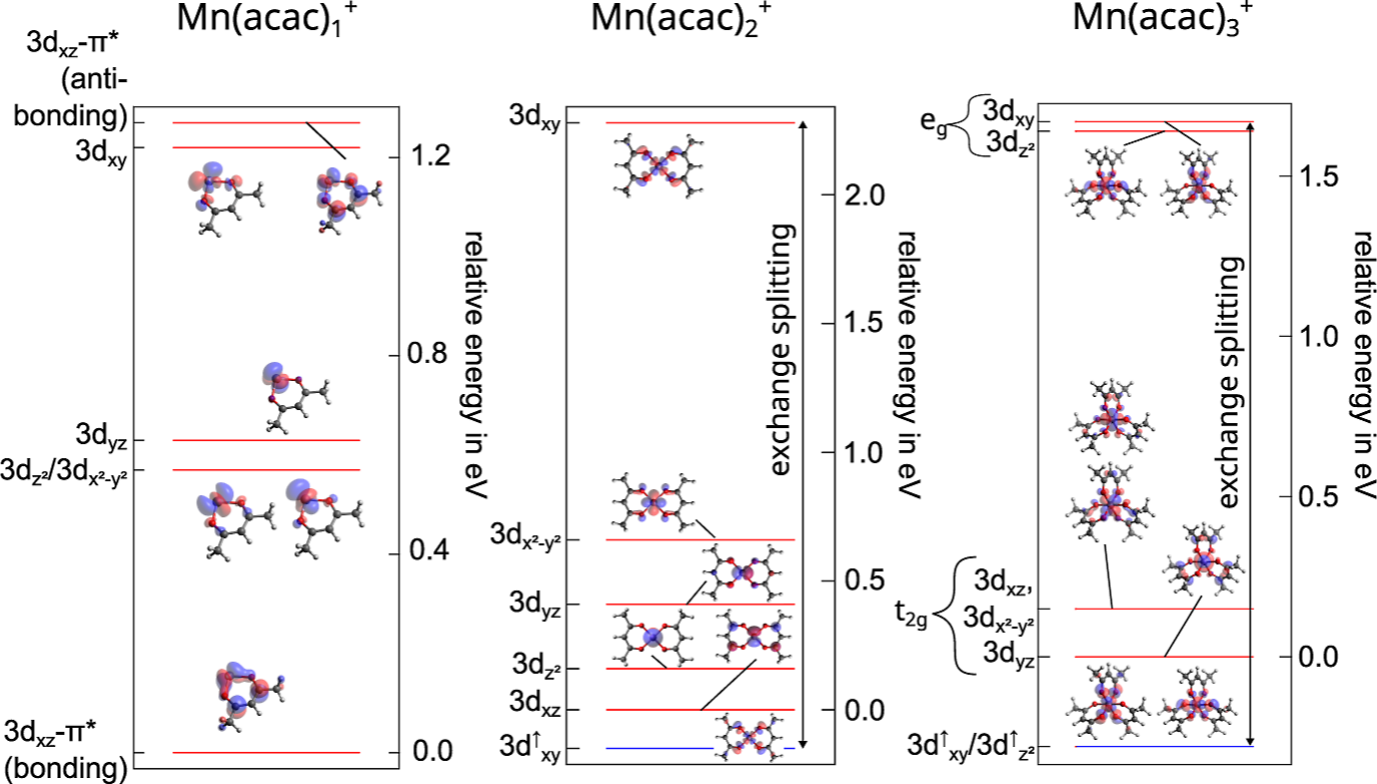
Energetic ordering of unoccupied molecular orbitals with significant Mn 3d or 4s character extracted from the oxygen K-edge of Mn(acac)_*n*_^+^, *n* = 1–3. Blue and red lines correspond to unoccupied spin up and down states, respectively. The origin of the relative energy scale is set to the lowest unoccupied minority spin state with significant 3d character. Isosurface plots with an isosurface value = 0.4 of these molecular orbitals are shown as well.

The good agreement of calculated and experimental spectra allows us to identify the molecular orbitals involved in the individual transitions represented as sticks in Figure 4. However, we are focusing only on those molecular orbitals that are hybrids of ligand^78^ and metal (manganese) 3d valence states.^48,79^ From the relative excitation energies into these molecular orbitals, we deduce an energetic ordering of the unoccupied 3d derived states as shown in Figure 5 along with isosurface plots of the respective molecular orbitals. All other transitions shown in Figure 4 are dominated by transitions into ligand type orbitals and are therefore omitted in Figure 5.

In the Mn(acac)_1_^+^ complex, the orbitals with strong 3d_*yz*_, , and  character group together within 0.2 eV being basically degenerate, while the 3d_*xy*_ orbital is pushed up in energy by about 0.6 eV. This is expected from a simple CF picture^48^ where the 3d_*xy*_ orbital has the highest density along the metal–oxygen bond. However, within the CF picture we would also expect the 3d_*xz*_ orbital to group with the 3d_*yz*_, , and  orbitals but instead the DFT calculation predicts a strong hybridization of the 3d_*xz*_ orbital with a ligand π* orbital resulting in the formation of bonding and antibonding molecular orbitals well separated in energy shifting the transitions related to the bonding and antibonding orbitals symmetrically down and up by about 0.6 eV, respectively. These transitions also lead to the largest discrepancies between the calculated and experimental spectra with an otherwise good agreement. Furthermore, the hybridization of the 3d_*xz*_ orbital is in contrast to the analysis of the Mn L_2,3_ edge spectra pointing to negligible hybridization of all the 3d states with the ligand orbitals. Hence, it seems that the strong hybridization of the 3d_*xz*_ orbital with ligand orbitals resulting in transitions at 531.4 and 532.6 eV are rather artifacts of the employed approximations to DFT that are known to over-delocalize 3d orbitals.^80^

In the Mn(acac)_2_^+^ complex, orbitals with , 3d_*yz*_, , and 3d_*xz*_ character are distributed over 0.7 eV instead of being tightly bunched together as in Mn(acac)_1_^+^. This is expected in a square planar symmetry as the degeneracy of the 3d_*yz*_, 3d_*xz*_, , and  orbitals is lifted by the lower symmetry of the system compared to the only weakly disturbed local spherical symmetry of Mn in Mn(acac)_1_^+^. Since, there is no ligand in *z*-direction, the  orbital should be lowest in energy experiencing the smallest influence of the ligand field of all the 3d orbitals. However, the orbital with 3d_*xz*_ character is slightly lower in energy by about 160 meV showcasing that the more subtle details of the electronic structure cannot be fully explained by CF theory alone but must also take into account the covalent bonding of the metal center to the ligands.

In the case of the Mn(acac)_3_^+^ complex, the orbitals group nicely into t_2g_ and e_g_ orbitals as expected^52,65,81^ in a local octahedral symmetry of the metal center. The average energy separation between the t_2g_ and e_g_ orbitals is 1.6 eV also indicated in Figure 4. A similar analysis of the oxygen K-edge has been applied to isoelectronic Cr(acac)_3_ in a condensed form, however, in contrast the t_2g_- and e_g_-like states could not be clearly disentangled and the splitting of the main resonance in the oxygen K-edge spectrum is only about 1 eV^81^ while it is 1.6 eV in the case of cationic Mn(acac)_3_^+^.

The overall grouping of the 3d states for all Mn(acac)_*n*_^+^ complexes, *n* = 1–3, is dominated by the CF as present in the respective systems and has been proposed before.^48^ Moreover, we are able to quantify the CF splitting as extracted from the experimental data by comparison to the TD-DFT calculations. Although different definitions could be applied and therefore result in slightly different absolute values, we follow the approach as outlined in ref (48), where the CF is defined as the energy separation of the 3d_*xz*,*yz*_ and 3d_*xy*_ orbitals. Since the 3d_*xz*_ and 3d_*yz*_ orbitals are not degenerate here, we opted for the average 3d_*xz*,*yz*_–3d_*xy*_ energy separation to quantify the CF splitting. Furthermore, in the case of Mn(acac)_2,3_^+^ we can additionally extract the exchange splitting from the oxygen K-edge spectrum as the energetic separation of 3d_*xy*_^↑^ and 3d_*xy*_^↓^ and energetically almost degenerate 3d_*xy*_^↑^, , 3d_*xy*_^↓^, and  states, respectively. This results in an exchange splitting of 2.4 eV for *n* = 2 and 2.0 eV for *n* = 3 as shown in Figure 5. These values are in line with rough estimates of an average value of 0.6 eV per unpaired spin.^77,82^ Again, the robustness of this approach is achieved by linking the experimental spectrum with the TD-DFT calculation to extract the energetic position of the unoccupied 3d orbitals. Extracting exchange and CF splittings from virtual orbitals alone could possibly not be very reliable as they strongly depend on the leftover basis functions and choice of the functional.

While showing a similar trend with increasing number of ligands, the values of the CF parameters extracted from the oxygen K-edge (0.6, 2.1, 1.6 eV) are systematically smaller than the 10*Dq* parameters extracted from the CF/CTM simulations (1.2, 2.4, 2.1 eV) of the manganese L_2,3_ edges. Deducing CF splittings directly from metal L-edge spectra is often hindered by the presence of multiplet splittings. However, in rare cases where the spectrum is dominated by CF splittings as for example in neutral Cr(acac)_3_^81^ they closely match the values extracted from oxygen K-edge spectra of isoelectronic Mn(acac)_3_^+^. Hence, showing that the 10*Dq* parameters, which are inputs to the CTM simulations, do not necessarily equal the apparent CF splittings as extracted from measured or simulated manganese L_2,3_ edge spectra. However, both show the same dependence on the number of ligands.

The electronic structure in Mn(acac)_*n*_^+^, *n* = 1–3, is dominated by a competition between CF strength and exchange energy. As discussed above, we can extract an exchange splitting from a comparison of experimental and TD-DFT-calculated oxygen K-edge spectrum, which is 2.4 and 2 eV in Mn(acac)_2,3_^+^, respectively, and is expected to be even larger in Mn(acac)_1_^+^. The CF splitting in Mn(acac)_1_^+^ is significantly smaller than the exchange energy resulting in an almost atomic-like X-ray absorption signal at the manganese L_2,3_ edges. In Mn(acac)_2,3_^+^, however, CF and exchange splitting are of the same magnitude, i.e., ∼2 eV. Hence, they have to be treated on the same footing making it more challenging to describe the electronic structure at the metal center correctly. Therefore, advanced multireference methods such as RASPT2 have to be employed to reproduce the spectral features satisfactorily.

### Carbon K-Edge Spectroscopy

**Figure 6:**
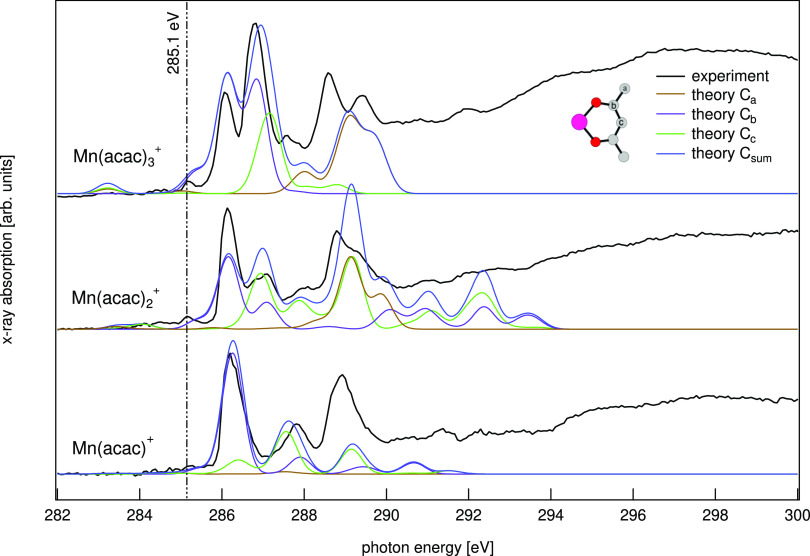
X-ray absorption spectra of gas-phase Mn(acac)_*n*_^+^, *n* = 1–3, at the carbon K-edge alongside TD-DFT calculations.

The last piece of information on the electronic structure results from the analysis of the carbon K-edge spectrum. In Figure 6, X-ray absorption spectra of gas-phase Mn(acac)_*n*_^+^, *n* = 1–3, at the carbon K-edge are shown and compared to theoretical spectra from TD-DFT calculations. We again focus our analysis at the low energies from 284 to 290 eV, where the spectrum can reliably be reproduced by the TD-DFT calculations.

Along the series there is a significant change of the leading low-energy line at about 286 eV, while higher-energy transitions are less affected. The low-energy transitions are dominated by transitions into orbitals with large contributions of the carbon atoms next to the metal–oxygen bridge (labeled C_b_ in the inset of Figure 6).

Similarly, in acetylacetone it has been shown that the leading line in the spectrum at about 286 eV is dominated by transitions into orbitals with C_b_ character and that the energetic position strongly depends on a chemical shift of the core 1s electrons induced by the proximity to the more electronegative oxygen atoms.^83^

The pre-peak at 285.1 eV highlighted by the dashed line in Figure 6 is the only transition at the carbon K-edge into orbitals with significant metal 3d character^69^ as inferred from a comparison to the TD-DFT calculations. The amount of metal 3d character in the valence orbitals decreases along the Mn(acac)_*n*_^+^ series, *n* = 1–3, substantiating the finding of an increase of the covalency of the metal–oxygen bond^84^ as discussed earlier.

## Conclusions

We studied and identified the electronic structure and oxidation state of cationic gas-phase Mn(acac)_*n*_^+^ complexes for the complete ligand series of *n* = 1–3 by combining XAS at the manganese L_2,3_, the oxygen as well as carbon K-edges with RAS/TD-DFT calculations and CF/CTM simulations. We find that in Mn(acac)_1_^+^ the electronic structure is dominated by exchange interaction with only a small influence of the ligands’ CF, while in Mn(acac)_2,3_^+^ exchange and CF splitting are competing and have therefore to be treated on the same footing. Overall our findings are in agreement with predictions.^48^ We have shown that empirical parameters such as CF strength and exchange splitting can not only be quantified by semi-empirical parametrized CF/CTM simulations or expensive wave function-based methods but also can be extracted from a comparison of experimental oxygen K-edge spectra and readily available TD-DFT calculations. This method can enable screening of metal complexes that might show exchange-enhanced reactivity^85^ or undergo spin-state changes induced by geometrical changes of reaction intermediates.^86^

Furthermore, we extracted oxidation states from L_3_ median excitation energy shifts for both cationic and neutral Mn(acac)_*n*_^0,+^. In the case of Mn(acac)_*n*_^0,+^ in the same oxidation state +2, we were able to disentangle the effects of varying 3d occupation^75^ and coordination.^76^ We show that this is a sizable effect of about 40–50% of the expected shift in L_3_ excitation energy per oxidation state^66,71^ and may be considered by spectroscopists employing L-edge shifts to determine oxidation states.

## Supporting Information Available

Ground-state structures for all complexes, additional details on RASSCF and RASPT2 calculations, comparison of calculated oxygen K-edge spectra for planar and distorted structure of Mn(acac)_2_^+^, systematic CF and CTM modeling for all complexes, photofragmentation channels and partial ion yields at all edges investigated, and details on determining L_3_ median excitation energy (PDF)
