# Supplementary material for: Electronic Structure of the Complete Series of Gas-Phase Manganese Acetylacetonates by X-ray Absorption Spectroscopy
Source: J Phys Chem A. 2023 Aug 17;127(34):7121–31. doi: 10.1021/acs.jpca.3c02794 (PMC10476195; doi:10.1021/acs.jpca.3c02794)
Supplement: Supplementary file 1 — jp3c02794_si_001.pdf [file jp3c02794_si_001.pdf]

Supplementary Information: Electronic Structure of the Complete Series of Gas-Phase Manganese Acetylacetonates by X-ray Absorption Spectroscopy

Olesya S. Ablyasova,[†,‡] Meiyuan Guo,[¶,§] Vicente Zamudio-Bayer,[‡] Markus Kubin,[‡] Tim Gitzinger,[†,‡] Mayara da Silva Santos,[†,‡] Max Flach,[†,‡] Martin Timm,[‡] Marcus Lundberg,[§] J. Tobias Lau,[†,‡] and Konstantin Hirsch[*,‡]

†*Physikalisches Institut, Albert-Ludwigs-Universität Freiburg, Hermann-Herder-Str. 3, 79104 Freiburg, Germany*

‡*Abteilung für Hochempfindliche Röntgenspektroskopie, Helmholtz-Zentrum Berlin für Materialien und Energie, Albert-Einstein-Str. 15, 12489 Berlin, Germany*

¶*SSRL, SLAC National Accelerator Laboratory, Menlo Park, California 94025, USA*

§*Department of Chemistry - Ångström Laboratory, Uppsala University, SE-75120 Uppsala, Sweden*

E-mail: Konstantin.Hirsch@helmholtz-berlin.de

Computational Details

Ground state structures of $Mn(acac)^{+}_{1-3}$ from DFT calculations

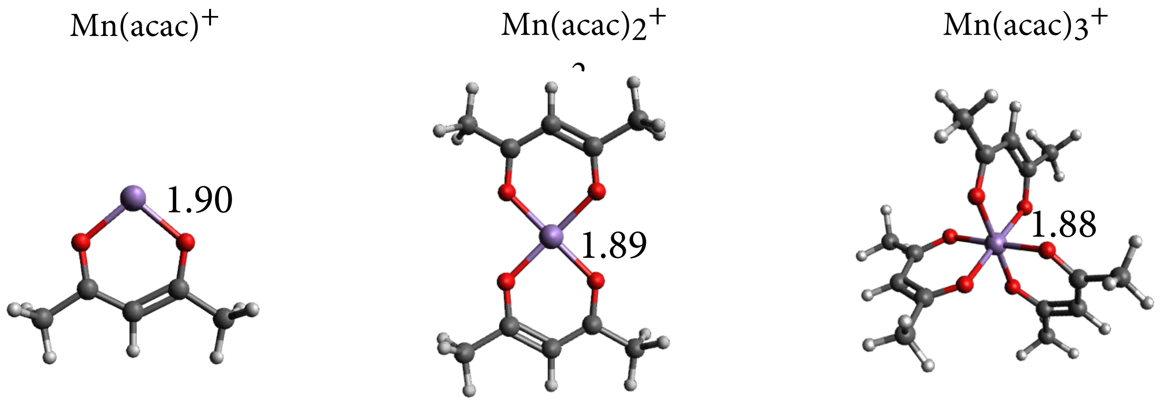

Figure S1: Ground state geometrical structures of $Mn(acac)^{+}_{n}$, $n = 1 - 3$ complexes optimized by B3LYP/6-31G(d) with Mn - O distance in Å. Atoms are depicted in the following colours: Mn: purple, O: red, C:grey, H: white.

$Mn(acac)_1^+$, multiplicity 2S+1=6

element	x	y	z
O	-0.000170	1.450390	0.492079
O	0.000170	-1.450390	0.492079
C	-0.000000	-1.260919	-0.800640
C	-0.000000	0.000000	-1.421851
H	-0.000000	0.000000	-2.504472
C	0.000000	1.260919	-0.800640
Mn	0.000000	-0.000000	1.719233
C	-0.000222	2.517202	-1.616716
H	0.000293	2.318110	-2.689255
H	-0.880555	3.117728	-1.358313
H	0.879342	3.118500	-1.357546
C	0.000222	-2.517202	-1.616716
H	-0.879342	-3.118500	-1.357546
H	-0.000293	-2.318110	-2.689255
H	0.880555	-3.117728	-1.358313

$Mn(acac)_2^+$, multiplicity 2S+1=5, planar structure

element	x	y	z
C	-3.354346	-2.521742	0.011332
H	-4.431302	-2.380620	0.014357
H	-3.057950	-3.101555	0.887862
H	-3.063800	-3.105389	-0.864701
C	-2.616243	-1.223890	0.005706
C	-3.281571	-0.000001	-0.000086
C	-2.616241	1.223888	-0.005723
C	-3.354344	2.521740	-0.011378
H	-3.056999	3.102130	-0.887191
H	-4.431296	2.380615	-0.015593
H	-3.064754	3.104811	0.865367
O	-1.327727	-1.331394	0.006184
O	-1.327727	1.331393	-0.005943
H	-4.359782	-0.000001	-0.000216
O	1.327725	-1.331393	-0.005971
O	1.327726	1.331393	0.006165
C	2.616240	1.223890	0.005745
C	3.281569	0.000001	-0.000015
H	4.359780	0.000001	-0.000086
C	2.616240	-1.223889	-0.005703
Mn	-0.000001	0.000000	0.000217
C	3.354344	-2.521740	-0.011377
H	4.431296	-2.380616	-0.015401
H	3.064603	-3.104919	0.865245
H	3.057148	-3.102023	-0.887314
C	3.354344	2.521741	0.011347
H	3.057537	3.101830	0.887550
H	4.431298	2.380618	0.014887
H	3.064211	3.105113	-0.865012

$Mn(acac)_2^+$, multiplicity 2S+1=5, distorted structure

element	x	y	z
C	-0.810646	-2.387293	3.351871
H	-0.771396	-2.248479	4.428291
H	-0.175925	-3.227062	3.063955
H	-1.828832	-2.645556	3.053466
C	-0.380352	-1.163754	2.612339
C	0.000006	-0.000005	3.276703
C	0.380350	1.163755	2.612347
C	0.810650	2.387288	3.351885
H	0.175932	3.227058	3.063970
H	0.771400	2.248471	4.428305
H	1.828837	2.645548	3.053479
O	-0.388745	-1.275293	1.323985
O	0.388726	1.275310	1.323994
H	0.000007	-0.000009	4.354364
O	-0.029386	-1.333047	-1.324015
O	0.029388	1.333052	-1.324022
C	0.009126	1.224434	-2.612446
C	-0.000000	-0.000003	-3.276616
H	-0.000002	-0.000006	-4.354287
C	-0.009126	-1.224437	-2.612440
Mn	0.000003	0.000004	0.000062
C	0.001337	-2.521533	-3.351791
H	0.006761	-2.377633	-4.428265
H	0.880916	-3.096374	-3.054961
H	-0.871466	-3.109704	-3.062702
C	-0.001338	2.521528	-3.351802
H	0.871465	3.109699	-3.062714
H	-0.006762	2.377627	-4.428276
H	-0.880917	3.096370	-3.054971

$Mn(acac)_3^+$, multiplicity 2S+1=4

element	x	y	z
C	0.00824500	1.22803600	2.59950100
C	0.00000000	0.00000000	3.26593700
C	-0.00824500	-1.22803600	2.59950100
O	0.02448900	1.35569600	1.31549800
O	-0.02448900	-1.35569600	1.31549800
H	0.00000000	0.00000000	4.34943100
O	0.00000000	-1.33640100	-1.34047400
O	-1.88915000	0.05168400	0.01668200
O	0.00000000	1.33640100	-1.34047400
O	1.88915000	-0.05168400	0.01668200
C	-1.00917200	-1.90673700	-1.90934500
C	-2.34380000	-1.59216900	-1.63820900
H	-3.11908800	-2.11850800	-2.17895800
C	-2.72139600	-0.62814300	-0.69932500
C	2.72139600	0.62814300	-0.69932500
C	2.34380000	1.59216900	-1.63820900
H	3.11908800	2.11850800	-2.17895800
C	1.00917200	1.90673700	-1.90934500
Mn	0.00000000	0.00000000	-0.00327200
C	4.16699100	0.30657100	-0.44592000
C	0.64275200	2.96065400	-2.91559600
C	-4.16699100	-0.30657100	-0.44592000
C	-0.64275200	-2.96065400	-2.91559600
C	0.00357500	2.52055800	3.37220400
C	-0.00357500	-2.52055800	3.37220400
H	1.52442400	3.41564100	-3.37015600
H	0.04371000	3.73656300	-2.42585900
H	0.01738500	2.51756400	-3.69875100
H	4.83380600	0.90245900	-1.07142300
H	4.34032100	-0.75802400	-0.63906200
H	4.40212900	0.48485300	0.60937400
H	-1.52442400	-3.41564100	-3.37015600
H	-0.04371000	-3.73656300	-2.42585900
H	-0.01738500	-2.51756400	-3.69875100
H	-4.83380600	-0.90245900	-1.07142300
H	-4.34032100	0.75802400	-0.63906200
H	-4.40212900	-0.48485300	0.60937400
H	-0.01036500	-3.37218500	2.69076600
H	-0.87972800	-2.57100600	4.02868900
H	0.88590600	-2.57314300	4.01025100
H	0.01036500	3.37218500	2.69076600
H	0.87972800	2.57100600	4.02868900
H	-0.88590600	2.57314300	4.01025100

Comparison of oxygen K-edge spectra of planar and distorted $Mn(acac)_2^+$ as calculated using TD-DFT

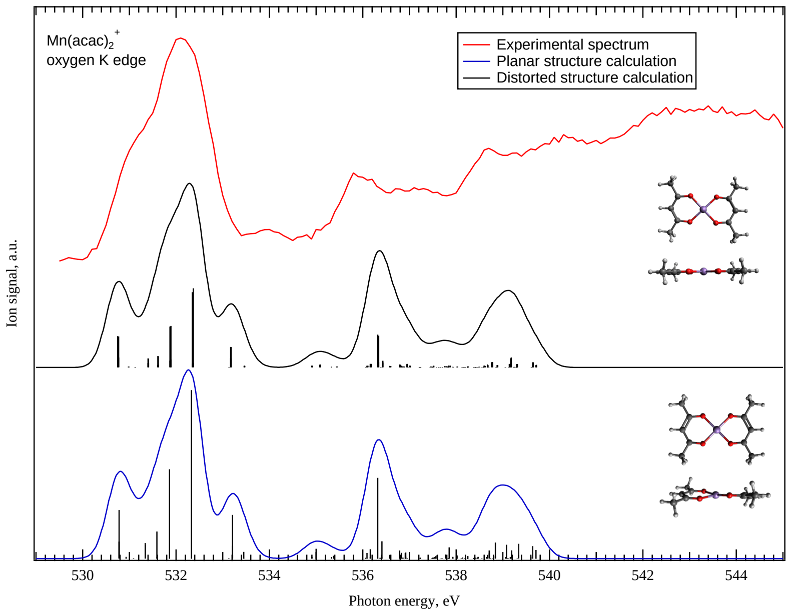

Figure S2: Comparison of the calculated X-ray absorption spectra at oxygen K edge of cationic $Mn^{III}(acac)_2^+$ in a planar and distorted geometry, which are energetically almost degenerate.

$L_{2,3}$-edge XAS from the RASPT2 calculations

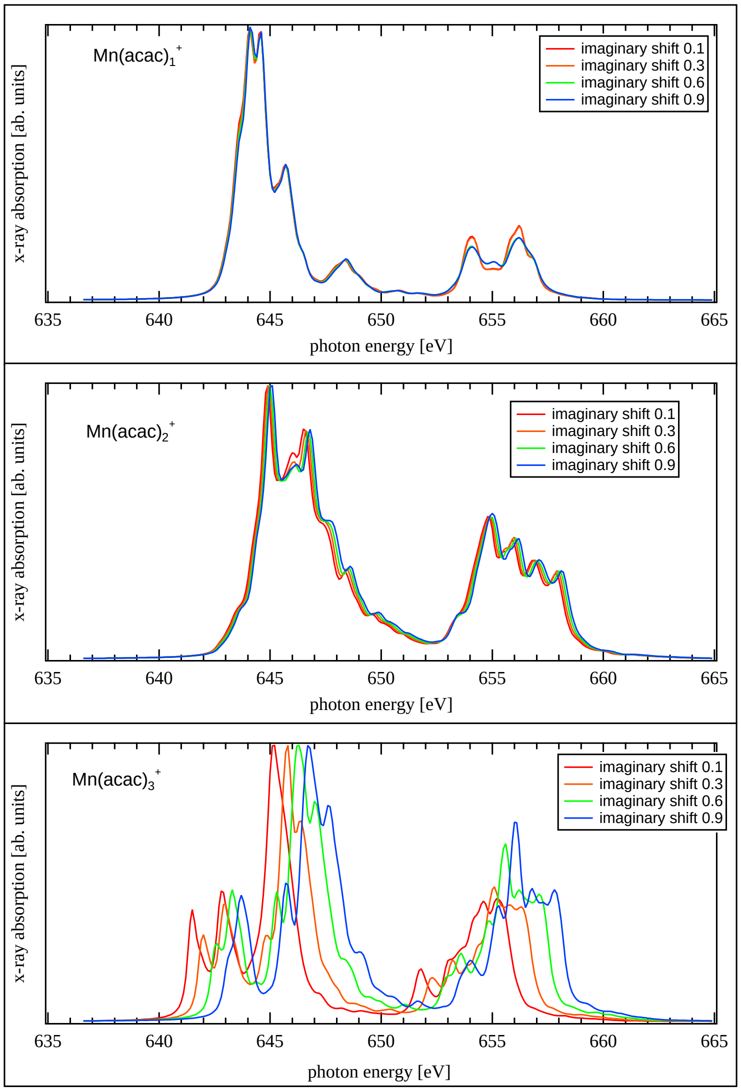

Figure S3: RASPT2 calculations of the $Mn(acac)_n^+$ ($n = 1 - 3$) varying the imaginary shifts from 0.1 to 0.9 hartree.

CFM and CTM simulations

Table S1: Parameters for crystal field and charge transfer multiplet (CTM) simulations of the X-ray absorption spectra at the manganese $L_{2,3}$-edges of $Mn(acac)^+_{1-3}$ complexes. For the CTM simulation of $Mn(acac)^+_3$ complex the parameters was taken from[1] for $Cr(acac)_3$ complex.

Complex	Symmetry	10Dq in eV	CFM parameters	CTM parameters in eV
$Mn(acac)^+_1$	C_{2v}	-1.2	$Dt=\frac{8}{7}Dq$, $Ds=-\frac{10}{7}Dq$	
$Mn(acac)^+_2$	D_{4h}	-2.4	$Dt=\frac{8}{7}Dq$, $Ds=-\frac{10}{7}$	$\Delta = 1$, $T_{b1} = T_{a1} = 0.5$, $T_{b2} = 1.5$, $T_e = 0.5$
$Mn(acac)^+_3$	O_h	2.1		$\Delta = 1$, $T_{eg} = 0.5$, $T_{t2g} = 0.25$, Q=U=1

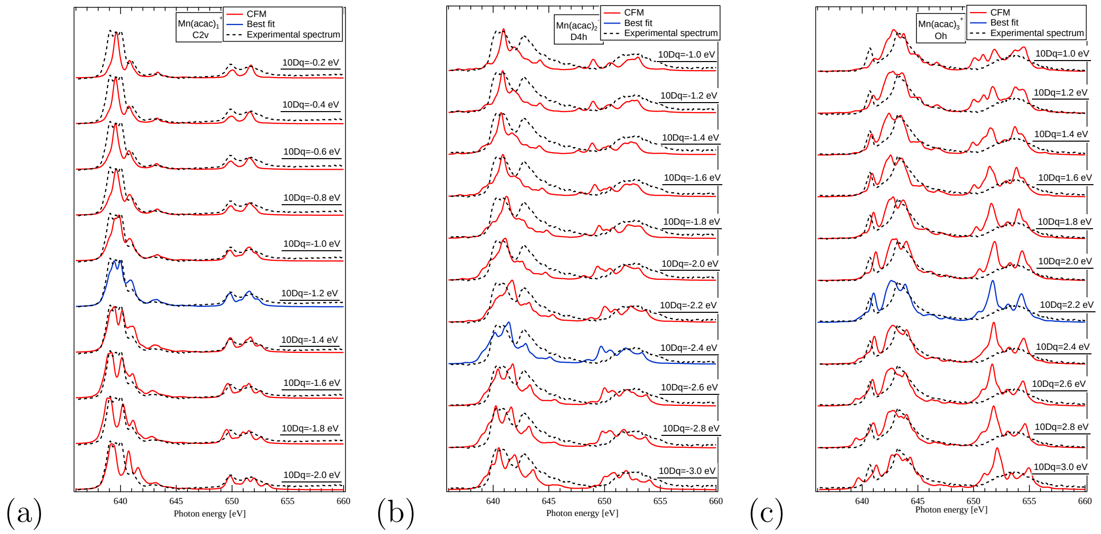

Figure S4: CF simulations for $Mn(acac)^+_n$ (a) n=1 (b) n=2 (c) n=3. The crystal field strength parameter 10Dq increases from top to bottom. In each panel the best match between simulation and experiment is shown in blue.

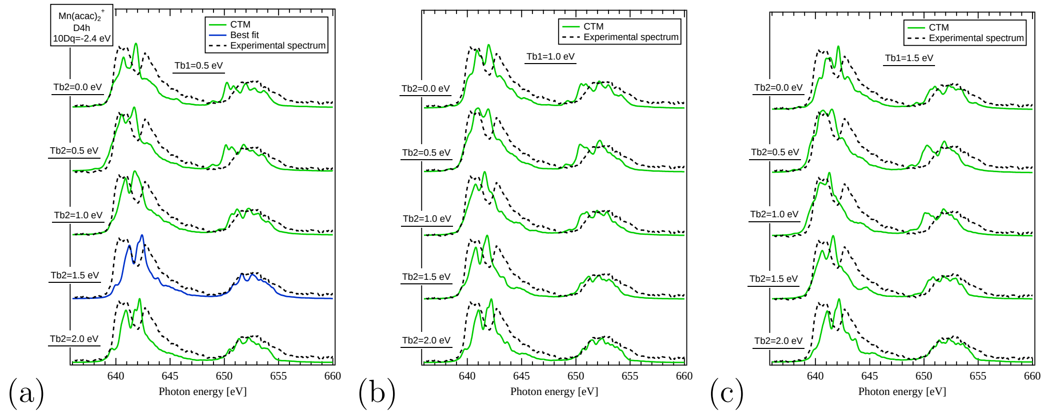

Figure S 5: CTM simulation for $Mn(acac)_2^+$using a fixed crystal field strength of 10Dq= -2.4 eV varying the hopping parameter T_{b2} for different values sets of fixed hopping parameters of (a) T_{b1}=T_{a1}== 0.5 eV, T_e=T_{b2} , (b) T_{b1}=T_{a1}=1.0 eV T_e=T_{b2}, and (c) T_{b1}=T_{a1}=1.5 eV, T_e=T_{b2}. The best match between simulation and experiment is shown in blue.

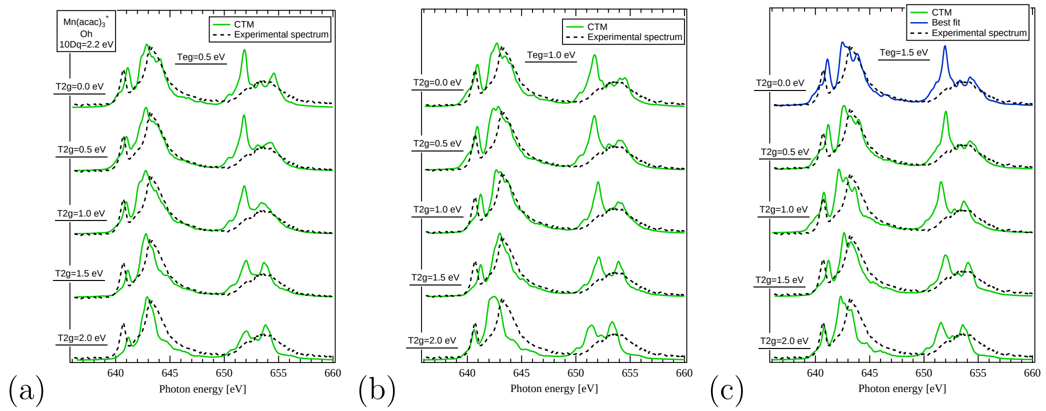

Figure S6: CTM simulation for $Mn(acac)_3^+$using a fixed value of the crystal field strength 10Dq= 2.2 eV varying the hopping parameter T_{t2g} for fixed values of (a) T_{eg} = 0.5 eV, (b) T_{eg} = 1.0 eV, and (c) T_{eg} = 1.5 eV. The best match between simulation and experiment is shown in blue.

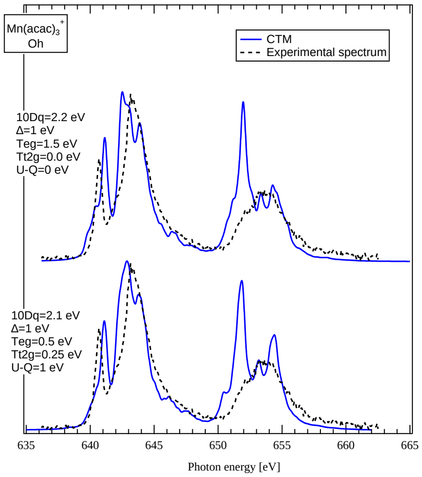

Figure S7: CTM simulation comparison for $Mn(acac)_3^+$ obtained in this paper and parameters proposed by Kubin[1] for isoelectronic $Cr(acac)_3$.

Experimental Details

Experimental Parameters

Table S2: Employed X-ray beam energy resolution and step size at the Mn $L_{2,3}$-edges, O K-edge, and C K-edge.

Complex	Mn $L_{2,3}$-edge		O K-edge		C K-edge	
	Energy resolution, meV	Step size, eV	Energy resolution, meV	Step size, eV	Energy resolution, meV	Step size, eV
$Mn(acac)_1^+$	170	0.08	200	0.1	100	0.07
$Mn(acac)_2^+$	160	0.08	200	0.1	100	0.05
$Mn(acac)_3^+$	180	0.08	235	0.14	100	0.07

Table S3: Energy shifts in eV applied to theoretical spectra at different levels of theory and edges with respect to experimental spectra of $Mn(acac)_n^+$, $n = 1 - 3$ complexes.

Complex	Mn $L_{2,3}$-edge RASSCF	Mn $L_{2,3}$-edge RASPT2	Mn $L_{2,3}$-edge CTM	O K-edge TD-DFT	C K-edge TD-DFT
$Mn(acac)_1^+$	-6.28	-4.56	-0.65	13.76	10.42
$Mn(acac)_2^+$	-6.57	-3.94	-0.54	14.44	10.36
$Mn(acac)_3^+$	-7.22	-2.13	-2.4	13.56	10.33

Photofragmention Channels

Table S4: Observed photofragments of $Mn(acac)^{+}_{1-3}$ complexes for X-ray excitation at the different absorption edges.

Complex	Mn $L_{2,3}$ edge	O K edge	C K edge
$Mn(acac)^{+}_{1}$	Mn^{+}	C_2^+, $C_2H_2^+$, $C_2H_3^+$, Mn^{++}, $C_2H_4^+$, COH^+, C_3^+, C_3H^+, $C_3H_2^+$, $C_3H_3^+$, C_2OH^+, $C_2OH_2^+$, $C_2OH_3^+$, Mn^+	C_2^+, $C_2H_2^+$, $C_2H_3^+$, Mn^{++}, $C_2H_4^+$, COH^+, C_3^+, C_3H^+, $C_3H_2^+$, $C_3H_3^+$, C_2OH^+, $C_2OH_2^+$, $C_2OH_3^+$, Mn^+
$Mn(acac)^{+}_{2}$	$C_2H_2^+$, $C_2H_3^+$, $C_2H_4^+$, COH^+, C_3^+, C_3H^+, $C_3H_2^+$, $C_3H_3^+$, C_2HO^+, $C_2H_2O^+$, $C_2H_3O^+$, C_4H^+, $C_4H_2^+$, $C_3HO_2^+$, Mn^+	$C_3H_2^+$, $C_3H_3^+$, C_2O^+, C_2HO^+, $C_2H_2O^+$, CO_2^+, CO_2H^+, $C_2H_3O^+$, C_4H^+, $C_4H_2^+$, $C_3HO_2^+$, Mn^+, $C_3OH_4^+$	$C_2H_2^+$, $C_2H_3^+$, $C_2H_4^+$, COH^+, C_3^+, C_3H^+, $C_3H_2^+$, $C_3H_3^+$, C_2O^+, C_2HO^+, $C_2H_2O^+$, CO_2^+, CO_2H^+, $C_2H_3O^+$, C_4H^+, $C_4H_2^+$, $C_3HO_2^+$, Mn^+, $MnO_2C_2^+$
$Mn(acac)^{+}_{3}$	$C_2H_2^+$, $C_2H_3^+$, $C_2H_4^+$, COH^+, COH_3^+, C_3H^+, $C_3H_2^+$, $C_3H_3^+$, C_2O^+, C_2HO^+, $C_2H_2O^+$, $C_2H_3O^+$, $C_2H_4O^+$, C_4H^+, $C_4H_2^+$, $C_4H_3^+$, C_3HO^+, $C_3H_2O^+$, Mn^+, $C_3H_4O_2^+$	$C_2H_2^+$, $C_2H_3^+$, $C_2H_4^+$, COH^+, COH_3^+, C_3H^+, $C_3H_2^+$, $C_3H_3^+$, C_2O^+, C_2HO^+, $C_2H_2O^+$, $C_2H_3O^+$, $C_2H_4O^+$, C_4H^+, $C_4H_2^+$, $C_4H_3^+$, C_3HO^+, $C_3H_2O^+$, Mn^+, $C_3H_4O_2^+$	$C_2H_2^+$, $C_2H_3^+$, $C_2H_4^+$, COH^+, COH_3^+, C_3H^+, $C_3H_2^+$, $C_3H_3^+$, C_2O^+, C_2HO^+, $C_2H_2O^+$, $C_2H_3O^+$, $C_2H_4O^+$, C_4H^+, $C_4H_2^+$, $C_4H_3^+$, C_3HO^+, $C_3H_2O^+$, Mn^+, $C_3H_4O_2^+$

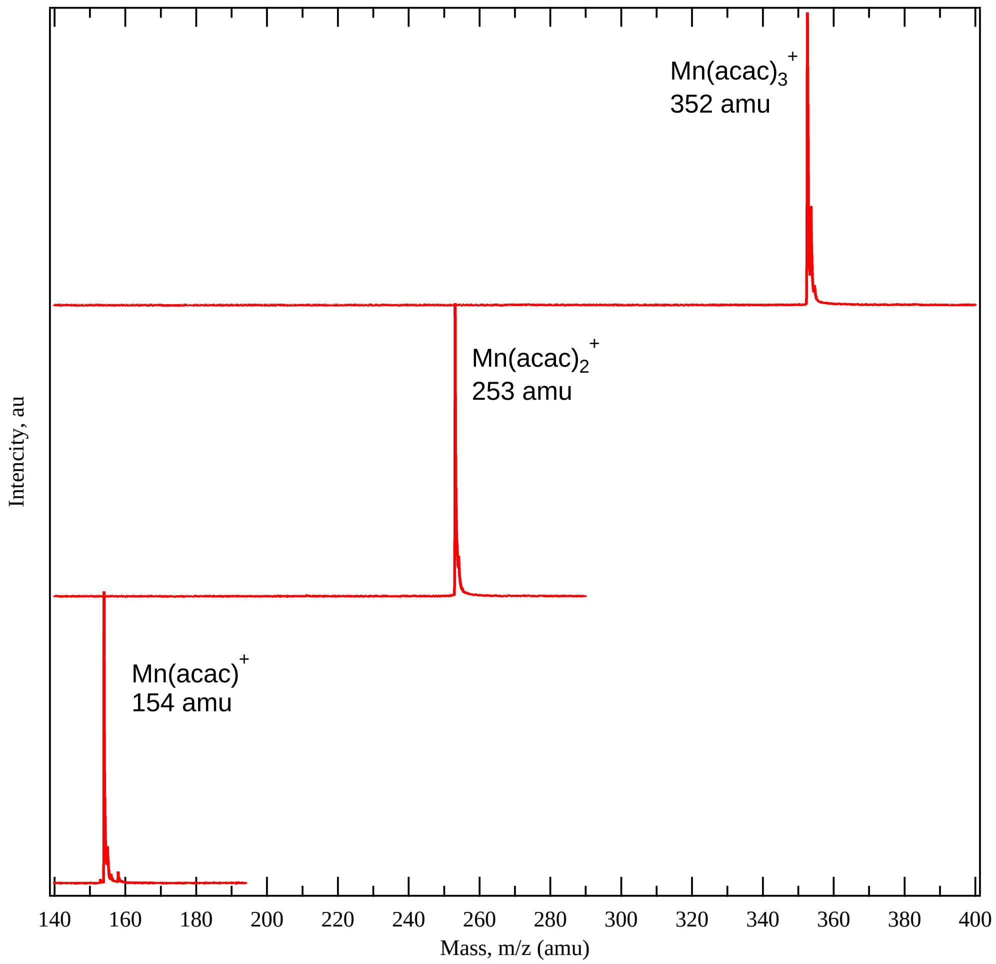

Figure S8: Time-of-flight mass spectra of trapped and mass-selected $Mn(acac)_{1-3}^{+}$ ions.

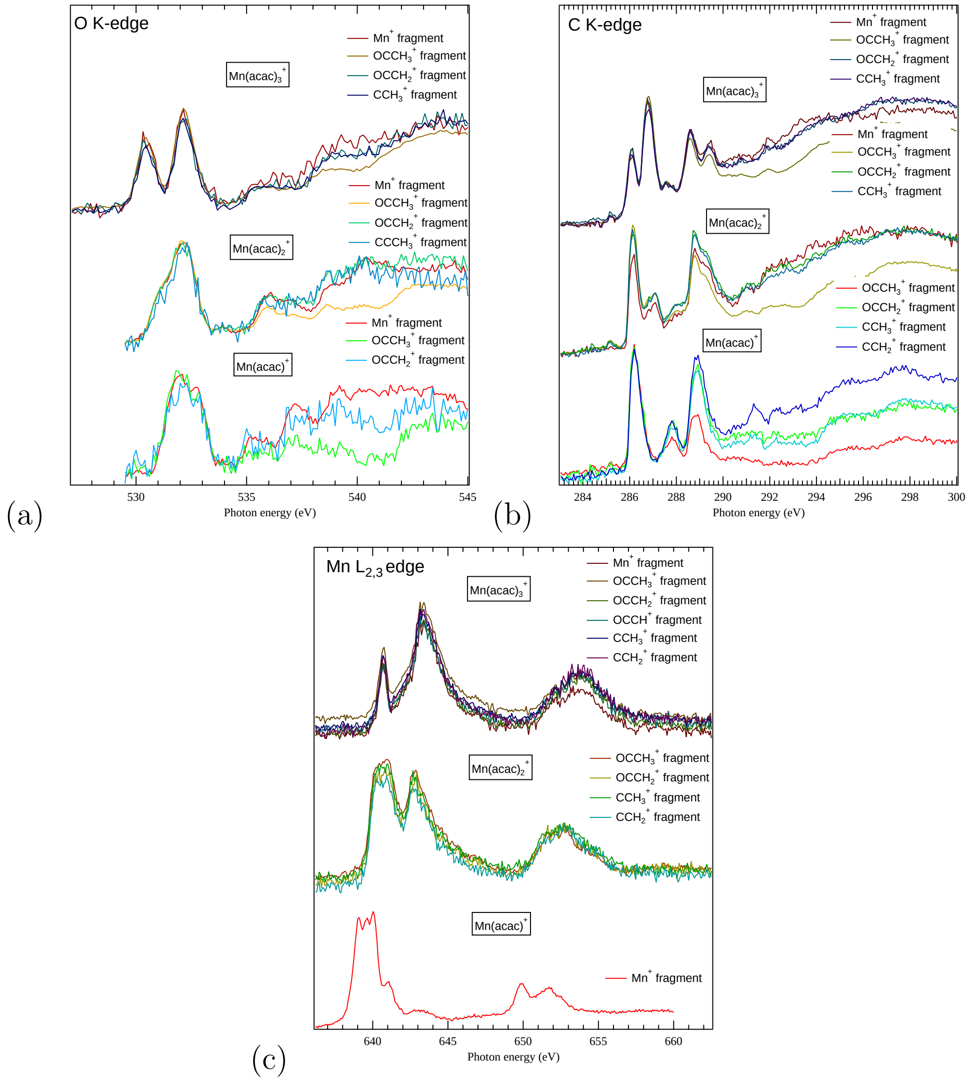

Figure S9: Ion yield spectra of the most intense fragments for $Mn(acac)_{1-3}^+$ parent complexes at (a) oxygen K-edge, (b) carbon K-edge, and (c) manganese $L_{2,3}$-edges.

Comparison of the peak splitting at the L_3 edge of $Mn^{III}(acac)_3$ and $Mn^{III}(acac)_2^+$

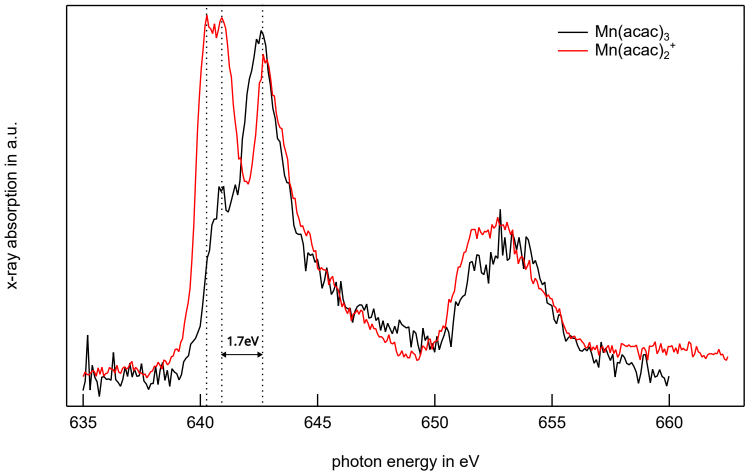

Figure S10: Comparison of the X-ray absorption spectra at manganese $L_{2,3}$ edges of neutral $Mn^{III}(acac)_3$[2] and cationic $Mn^{III}(acac)_2^+$. Despite distinctively different spectral shapes the splitting of the L_3 line by 1.7 eV is similar in both cases. The spectrum of neutral $Mn^{III}(acac)_3$ has been shifted by 1.07 eV to highlight the comparable splitting at the L_3 edge.

Determination L_3 median excitation energy

Table S5: Oxidation state assignment and median L_3 excitation energy extracted from experimental and theoretical Mn L-edge x-ray absorption spectra for cationic $Mn(acac)_n^+$ ($n = 1 - 3$) and neutral $Mn(acac)_n$ ($n = 2, 3$) complexes. Also given are the slope and the offset of the respective linear fits of the L_3 median excitation energy as a function of formal oxidation state

$Mn(acac)_n^{0,+}$ experiment				
Complex	Oxidation state	Median position, eV	Slope, eV / oxidation state	Offset, eV
$Mn(acac)_1^+$	+2	639.80 ±0.17		
$Mn(acac)_2^+$	+3	642.08 ±0.20	1.9 ±0.1	636.17
$Mn(acac)_3^+$	+4	643.56 ±0.18		
$Mn(acac)_2$	+2	640.6 ±0.3		
$Mn(acac)_3$	+3	641.8 ±0.3	1.3	638.2
$Mn(acac)_n^{0,+}$ RAS calculation				
Complex	Oxidation state	Median position, eV	Slope, eV / oxidation state	Offset, eV
$Mn(acac)_1^+$	+2	646.32		
$Mn(acac)_2^+$	+3	647.95	2.22 ±0.34	641.7
$Mn(acac)_3^+$	+4	650.75		
$Mn(acac)_2$	+2	644.92		
$Mn(acac)_3$	+3	646.5	1.58	641.8

In order to extract the median energy for resonant excitations in the vicinity of the L_3 absorption edge, which is known to correlate with the oxidation state of the system, we need to subtract the contributions of the direct photoionization of the 2p electrons first. The total uncertainty was obtained through error propagation taking into account the uncertainties of beamline and uncertainties introduced by integration limits analogue to the procedure applied in Ref.[3] All values are given in SI table 5.

References

(1) Kubin, M.; Guo, M.; Ekimova, M.; Källman, E.; Kern, J.; Yachandra, V. K.; Yano, J.; Nibbering, E. T. J.; Lundberg, M.; Wernet, P. Cr L-Edge X-ray Absorption Spectroscopy of CrIII(acac)3 in Solution with Measured and Calculated Absolute Absorption Cross Sections. *J. Phys. Chem. B* **2018**, *122*, 7375–7384.

(2) Kubin, M.; Guo, M.; Kroll, T.; Löchel, H.; Källman, E.; Baker, M. L.; Mitzner, R.; Gul, S.; Kern, J.; Föhlisch, A. et al. Probing the oxidation state of transition metal complexes: a case study on how charge and spin densities determine Mn L-edge X-ray absorption energies. *Chem. Sci.* **2018**, *9*, 6813–6829.

(3) Flach, M.; Hirsch, K.; Timm, M.; Ablyasova, O. S.; Santos, M. d. S.; Kubin, M.; Bülow, C.; Gitzinger, T.; von Issendorff, B.; Lau, J. T. et al. Iron L3-edge Energy Shifts for the Full Range of Possible 3d Occupations within the Same Oxidation State of Iron Halides. *Phys. Chem. Chem. Phys.* **2022**, *24*, 19890–19894.
